# MOTIVATING CHANGE IN OLDER ADULTS: MOTIVATIONAL CIGARETTE SMOKING CESSATION MESSAGE TESTING

**DOI:** 10.1093/geroni/igac059.2713

**Published:** 2022-12-20

**Authors:** Adrienne Johnson, Jesse Kaye

**Affiliations:** University of Wisconsin School of Medicine and Public Health - Center for Tobacco Research and Intervention, Madison, Wisconsin, United States; University of Wisconsin School of Medicine and Public Health - Center for Tobacco Research and Intervention, Madison, Wisconsin, United States

## Abstract

Cigarette smoking remains the leading preventable cause of death and disability in the U.S., resulting in ~480,000 deaths annually. Older adults who smoke bear a disproportionate weight of the health consequences of smoking, including cancer, mortality, and the greatest health-related fear of older adults: dementia. Compared to younger adults, older adults who smoke are half as likely to make a quit attempt, but more likely to stay quit using evidence-based treatments. Research suggests the increased risk of dementia among people who currently smoke may motivate adults ages >50 to quit smoking, particularly if given a clear/actionable strategy. Research also suggests Fear-based messages may perform differently than Hope-based messages. 820 adults (ages 50–80) without dementia who smoke, completed an online survey evaluating time-matched messages (randomly assigned between-subjects: Control Nf266, Fear of dementia Nf274, Hope from quitting Nf280) on motivation and intentions to quit smoking. Participants’ demographics were Mage=61.1 years (SD=7.4), 48.0% cisgender women, 66.6% White, 23.3% Black. Mann-Whitney U Tests were use to examine change scores for each variable due to non-normal distributions. Compared to control message (water ad), the Fear message showed greater increase in motivation to quit U(Ncontrol=266, Nfear=274)=30391, z=-3.33, p=.001. The Hope message did not differ from the control or Fear message (p’s>.05). Intention to quit did not differ between messages (p’s>.05). A Fear-based message highlighting that smoking increases the risk of developing dementia, motivated quitting more than a control message. Future work should examine the feasibility, acceptability, and behavioral impact of this motivational message in healthcare settings.

